# Letter to the editor in response to the Japanese clinical practice guidelines for rehabilitation in critically ill patients 2023 (J-ReCIP 2023)

**DOI:** 10.1186/s40560-024-00732-4

**Published:** 2024-06-27

**Authors:** Charissa J. Zaga, Sarah Wallace, Amy Freeman-Sanderson

**Affiliations:** 1 Department of Speech Pathology, Division of Allied Health, Austin Health, Melbourne, Australia; 2 Department of Audiology and Speech Pathology, Faculty of Medicine, Dentistry and Health Sciences, The University of Melbourne, Melbourne, Australia; 3 Department of Critical Care, Faculty of Medicine, Dentistry and Health Sciences, The University of Melbourne, Melbourne, Australia; 4 Implementation Science Unit, Institute for Breathing and Sleep, Austin Health, Melbourne, Australia; 5 Graduate School of Health, University of Technology Sydney, Ultimo, NSW Australia; 6 Royal Prince Alfred Hospital, Sydney, NSW Australia; 7 Critical Care Division, The George Institute for Global Health, Faculty of Medicine, UNSW Sydney, Sydney, Australia; 8 Australian and New Zealand Intensive Care Research Centre (ANZIC-RC), School of Public Health and Preventive Medicine, Monash University, Melbourne, Australia; 9 Department of Speech Voice and Swallowing, Wythenshawe Hospital, Manchester University NHS Foundation Trust, Manchester, UK; 10 Division of Infection Immunity and Respiratory Medicine, University of Manchester, Manchester, UK

**Dear Editor**,

We applaud Unoki et al. on their recent development of the Japanese clinical practice guidelines (CPGs) for rehabilitation in critically ill patients 2023 (J-ReCIP 2023). These aim to advocate for early initiation of rehabilitation in Japanese Intensive Care Units (ICU) to optimize patient outcomes. Whilst a rigorous methodology were used, evidence informing these guidelines were limited to RCTs and failed to consider important evidence offered by other research designs on assessment of swallowing function. As Speech Pathologists, our comments relate specifically to the WG3 Dysphagia and the recommendation made against the use of videoendoscopic swallowing assessment.

We agree that the exact prevalence of dysphagia is uncertain, with reports up to 91% [1]. Dysphagia in critical illness is a common issue with multiple negative long term sequelae and is significantly associated with an increased risk of mortality [2, 3]. Accurate and comprehensive assessment of swallow function, opposed to screening in isolation, is key to evidence-based informed management. The authors report swallowing function is often impaired due to interventions such as the placement of endotracheal and tracheostomy tubes and other surgical procedures. These aetiologies extend further to include both comorbidities and current acute medical diagnoses with their associated complications. Specifically, diagnosed sepsis, critical illness neuromyopathy, reflux, altered ventilation status, and impaired cognition can impair both the safety and efficiency of swallow function [4, 5]. Early rehabilitation in this population should optimize sensory and motor function with interventions including laryngeal re-sensitization and restoration of airflow through the upper airway to promote sensation, swallow, cough, smell and communication.

While swallow screening in the ICU is an initial step for identifying dysphagia, few existing screening tools have been validated for use in the critical care setting and importantly, screening is not equivalent to comprehensive assessment. Attachment to medical devices and lack of mobility does not preclude swallowing assessment in ICU. Commonly, Speech Pathologists will commence intervention with the clinical swallowing evaluation (CSE), which includes oral peripheral and cranial nerve examinations, assessment of upper airway function and suitability of oral trials to determine the presence, severity, and pathophysiology of dysphagia. Significant limitations exist with the CSE, as subjective inferences are made which are not grounded in strong evidence. As the authors highlighted, silent aspiration is common in critically ill patients and difficult to detect via CSE. Therefore, the gold standard instrumental assessments of Flexible Endoscopic Evaluation of Swallowing (FEES) (referred to as videoendoscopic examination of swallowing in Unoki et al.’s article) and videofluoroscopic swallowing studies offer paramount diagnostic accuracy [4]. As FEES is conducted at the bedside, it is ideally suited to the ICU setting and shown to be both safe and efficacious [6]. These assessments uniquely offer assessment of (1) presence and degree of laryngeal penetration and aspiration; (2) response to airway compromise (no response, silent laryngeal penetration or silent aspiration, successful or unsuccessful attempt to eject the bolus from the airway); (3) the dynamic swallowing profile across oral, pharyngeal and upper oesophageal domains; (4) swallow timing, motor and sensory functions; (5) presence and degree of bolus residue; (6) impact of structural changes on the swallow function (e.g., vocal cord palsy); (7) secretion management and the impact on airway protection; (8) targeted physiology-based rehabilitation planning. FEES also provides detailed information from visualization of laryngeal injury following intubation or other comorbidities, which can impact multidisciplinary tracheostomy weaning and decannulation decisions. The authors report the findings of Barquist et al.’s [7] study as increased harm (defined as increased aspiration) in the intervention group. It is important to consider whilst there was more aspiration noted, aspiration was not associated with FEES, but rather FEES was more responsive to detecting harms, hence the increased prevalence. As aspiration can lead to pneumonia, early detection of harm (i.e. aspiration) is vital to mitigate further morbidity and mortality risk, and in turn can optimise patient safety.

We therefore strongly disagree with the recommendation against using videoendoscopic examination of swallowing to manage critically ill patients. Our opinion is that FEES is an essential component of assessment and management of swallowing, and this is upheld by guidelines of Peak National Associations [8]. FEES can expedite the commencement of safe oral intake due to its accuracy in detection of aspiration risk [9] over other assessment methods [6]. Commencement of earlier safe oral feeding is crucial to improvement in mood, nutrition and engagement in physical rehabilitation. Therefore, the recommendation against utilizing FEES seems in direct opposition to the goal of diagnostic accuracy for evidence informed rehabilitation. Indeed, a suboptimal swallow assessment is potentially of greater harm due to the known sequelae of morbidity, cost, and mortality of dysphagia.

We agree that future research is warranted to further determine the prevalence of dysphagia, and there is an urgency to guide the optimal approach to dysphagia assessment, distinguishing screening from clinical assessment. Whilst we acknowledge that the evidence could be strengthened further in the rehabilitation context, we urge the authors to reconsider their position on FEES and make a conditional recommendation advocating for dysphagia management based on videoendoscopic examination in the critically ill.

## Data Availability

Not applicable.

## References

[1] Ponfick M, Linden R, Nowak DA (2015). Dysphagia—a common, transient symptom in critical illness polyneuropathy: a fiberoptic endoscopic evaluation of swallowing study. Crit Care Med.

[2] Zuercher P, Moser M, Waskowski J, Pfortmueller CA, Schefold JC (2022). Dysphagia post-extubation affects long-term mortality in mixed adult ICU patients—data from a large prospective observational study with systematic dysphagia screening. Crit Care Explor.

[3] Likar R, Aroyo I, Bangert K, Degen B, Dziewas R, Galvan O, Grundschober MT, Köstenberger M, Muhle P, Schefold JC, Zuercher P (2024). Management of swallowing disorders in ICU patients-a multinational expert opinion. J Crit Care.

[4] Brodsky MB, Pandian V, Needham DM (2020). Post-extubation dysphagia: a problem needing multidisciplinary efforts. Intensive Care Med.

[5] Zuercher P, Moret CS, Dziewas R (2019). Dysphagia in the intensive care unit: epidemiology, mechanisms, and clinical management. Crit Care.

[6] Morris K, Taylor NF, Freeman-Sanderson A (2024). Safety-related outcomes for patients with a tracheostomy and the use of flexible endoscopic evaluation of swallowing (FEES) for assessment and management of swallowing: A systematic review. Int J Speech Lang Pathol.

[7] Barquist E, Brown M, Cohn S, Lundy D, Jackowski J (2001). Postextubation fiberoptic endoscopic evaluation of swallowing after prolonged endotracheal intubation: a randomized, prospective trial. Crit Care Med.

[8] The Faculty of Intensive Care Medicine & Intensive Care Society. Guidelines for the Provision of Intensive Care Services v2.1. 2022; https://www.ficm.ac.uk/standards/guidelines-for-the-provision-of-intensive-care-services.

[9] Dziewas R, Aufdem Brinke M, Birkmann U, Bräuer G, Busch K, Cerra F, Damm-Lunau R, Dunkel J, Fellgiebel A, Garms E, Glahn J, Hagen S, Held S, Helfer C, Hiller M, Horn-Schenk C, Kley C, Lange N, Lapa S, Ledl C, Lindner-Pfleghar B, Mertl-Rötzer M, Müller M, Neugebauer H, Özsucu D, Ohms M, Perniß M, Pfeilschifter W, Plass T, Roth C, Roukens R, Schmidt-Wilcke T, Schumann B, Schwarze J, Schweikert K, Stege H, Theuerkauf D, Thomas RS, Vahle U, Voigt N, Weber H, Werner CJ, Wirth R, Wittich I, Woldag H, Warnecke T (2019). Safety and clinical impact of FEES—results of the FEES-registry. Neurol Res Pract.

